# Formulation and Characterization of Electrospun Nanofibers for Melatonin Ocular Delivery

**DOI:** 10.3390/pharmaceutics15041296

**Published:** 2023-04-20

**Authors:** Alessia Romeo, Adrienn Kazsoki, Safaa Omer, Balázs Pinke, László Mészáros, Teresa Musumeci, Romána Zelkó

**Affiliations:** 1 Department of Drug and Health Sciences, Laboratory of Drug Delivery Technology, University of Catania, Viale A. Doria 6, 95125 Catania, Italy; alessia.romeo@phd.unict.it (A.R.);; 2 University Pharmacy Department of Pharmacy Administration, Semmelweis University, Högyes Endre utca 7-9, H-1092 Budapest, Hungary; kazsoki.adrienn@pharma.semmelweis-univ.hu (A.K.);; 3 Department of Polymer Engineering, Faculty of Mechanical Engineering, Budapest University of Technology and Economics, Műegyetem rkp. 3, H-1111 Budapest, Hungary; 4 NANOMED—Research Centre for Nanomedicine and Pharmaceutical Nanotechnology, Department of Drug and Health Sciences, University of Catania, Viale A. Doria 6, 95125 Catania, Italy

**Keywords:** nanofibers, ocular insert, electrospinning, PLA, PVA, in vitro dissolution study, ocular delivery

## Abstract

The poor ocular bioavailability of melatonin (MEL) limits the therapeutic action the molecule could exert in the treatment of ocular diseases. To date, no study has explored the use of nanofiber-based inserts to prolong ocular surface contact time and improve MEL delivery. Here, the electrospinning technique was proposed to prepare poly (vinyl alcohol) (PVA) and poly (lactic acid) (PLA) nanofiber inserts. Both nanofibers were produced with different concentrations of MEL and with or without the addition of Tween^®^ 80. Nanofibers morphology was evaluated by scanning electron microscopy. Thermal and spectroscopic analyses were performed to characterize the state of MEL in the scaffolds. MEL release profiles were observed under simulated physiological conditions (pH 7.4, 37 °C). The swelling behavior was evaluated by a gravimetric method. The results confirmed that submicron-sized nanofibrous structures were obtained with MEL in the amorphous state. Different MEL release rates were achieved depending on the nature of the polymer. Fast (20 min) and complete release was observed for the PVA-based samples, unlike the PLA polymer, which provided slow and controlled MEL release. The addition of Tween^®^ 80 affected the swelling properties of the fibrous structures. Overall, the results suggest that membranes could be an attractive vehicle as a potential alternative to liquid formulations for ocular administration of MEL.

## 1. Introduction

Oxidative cellular stress is one of the main risk factors in the onset of neurodegenerative ocular diseases. The inflammatory processes that occur as a result of an oxidative insult trigger a series of events such as protein modifications, DNA damage and induction of apoptosis, which ultimately cause the death of neurons [1]. These phenomena underlie the pathogenesis of neurodegenerative diseases. Antioxidant and anti-inflammatory substances could be a useful treatment option to restore and protect neurons exposed to pathogenicity conditions [2]. Melatonin (MEL; N-acetyl-5-methoxytriptamine) is an indole heterocyclic compound synthesized from serotonin. Secretion of this neurohormone mainly takes place in the pineal gland, but also in extrapineal structures such as the iris, lens, ciliary body, and retina [3]. Therefore, over the past decades, several studies have investigated the therapeutic role of MEL in neurodegenerative ocular disorders. The antioxidant, anti-inflammatory, immunomodulatory and neuroprotective properties of MEL in the ocular structure have been demonstrated both in animal models and in clinical studies [4,5]. Reduction of oxidative stress, inhibition of the expression of pro-inflammatory cytokines (IL-6 and TNF-α) and angiogenic growth factor (VEGF) are some of the mechanisms whereby MEL exerts its neuroprotective effect against retinal injury and neuronal damage associated to several chronic and degenerative ocular diseases such as age-related macular degeneration (AMD), diabetic retinopathy (DR) and glaucoma [6,7]. However, most clinical studies for the treatment of ocular disorders are based on MEL oral administration [6,8]. On the other hand, the short plasma half-life and low bioavailability (3–15%) pose a limit to therapeutic efficacy [9,10]. For the treatment of chronic disorders, frequent drug doses are required to maintain effective treatment regimes. For this purpose, different nanotechnologies with mucoadhesive and mucopenetrating properties (nanocapsules, liposomes, micelles, and nanoparticles) have been investigated to improve corneal penetration and enhance MEL delivery in intraocular tissues [11,12,13,14,15,16,17]. To date to the best of our knowledge, no nanofibre-based device has been investigated to improve ocular bioavailability of MEL. Electrospinning is a top-down, efficient, easy and versatile process that has gained much attention in the last decade for the fabrication of nanofibers [18]. This technique allows obtaining polymeric nanofibers with diameters in the nanometric range from polymeric solutions and melts. Among the commonly used polymers for electrospun nanofibers are polylactic acid (PLA), poly(lactic-co-glycolic acid) PLGA, polyvinyl alcohol (PVA) and polyvinylpyrrolidone (PVP). The interesting mechanical, chemical and electrical properties of polymers are useful to obtain resorbable devices with flexible, porous structures and a high surface-to-volume ratio [19,20]. All of them are FDA-approved for use in formulations intended for ocular application [21].

The enormous advantages of biodegradable polymers combined with those of electrospun nanofibers make nanofiber-based inserts promising ocular drug delivery systems. The positioning of the scaffolds in the conjunctival sac bypasses the precorneal barriers (rapid tear fluid turnover and nasolacrimal drainage) typically associated with topical instillation of eye drops [22]. The softness and elasticity of nanofibrous implants ensure an easy, comfortable and prolonged fit on the surfaces of the cornea and sclera [23]. So, the advantages of these innovative delivery systems are many, such as the prolonged retention time which promotes transcorneal drug absorption, the reduced frequency of administration which improves patient compliance, and the absence of nasolacrimal absorption which moderates systemic side effects [24].

The aim of this study was to formulate and characterize for the first time MEL-loaded nanofibers for potential ocular administration. Hydrophobic PLA and hydrophilic PVA polymers were selected to develop electrospun nanofibers. Both nanosystems were loaded with different concentrations of MEL (0.1, 0.3 and 0.5% *w*/*w*). Tween^®^ 80 as permeation enhancer was investigated for all nanofibers to observe variations in the final properties of the nanosystems compared to the ones obtained without the permeation enhancer. The structural characterization of formulated fibrous inserts was in the focus of this work. Differential Scanning Calorimetry (DSC) and Fourier-transform Infrared Spectroscopy (FT-IR) were performed to characterize the solid-state samples. The morphology of the composite nanofibers was investigated by scanning electron microscope (SEM). Release profiles were measured under simulated physiological conditions (Phospate Buffer Saline (PBS) at pH 7.4, 37 °C). The swelling degree (%SD) of the produced nanofibers was also analyzed.

## 2. Materials and Methods

### 2.1. Materials

Melatonin (powder <98% (TLC), Cas number: 73-31-4, Mw: 232.28), polyvinyl alcohol (PVA, Mowiol^®^ 18–88, average molecular weight Mw ~130.000 g mol^−1^), polylactic acid, chloroform (Chl, Anhydrous, contains amylenes as stabilizer, <99%) and N,N-dimethylformamide (DMF, Anhydrous, 99.8%) were obtained from Sigma-Aldrich (Budapest, Hungary). Ethanol 96%, Polysorbatum 80 (Tween^®^ 80), potassium dihydrogen phosphate and sodium hydroxide were purchased from Molar Chemicals (Budapest, Hungary). Materials were used without additional purification and deionized water was of analytical grade.

### 2.2. Preparation and Electrospinning of PLA Nanofibers

The electrospinning process was performed at 7% (*w*/*w*) PLA polymer concentration, using binary solvent system to solubilize the hydrophobic polymer. Briefly, PLA polymer was dissolved in Chl:DMF in a 6:1 mass ratio and stirred under magnetic stirring until a clear and viscous solution was obtained. To produce empty-PLA nanofibers, electrospinning processes studies were conducted at different voltages and flow rates. Voltages were varied from 10 to 15 kV, while the flow rates were investigated from 0.2 to 0.5 µL/s. All the fibers were collected on aluminum foil, and the distance between the needle and the collector was fixed at 20 cm. To obtain drug-loaded PLA nanofibers, different concentration of MEL (0.1, 0.3 and 0.5% *w*/*w*) were blended into PLA polymer solution with 7% (*w*/*w*) PLA concentration with and without Tween^®^ 80 (0.5% *w*/*w*). The composition of the PLA-based nanofiber samples and the amounts of the components applied are described in Table 1.

**Figure 1:**
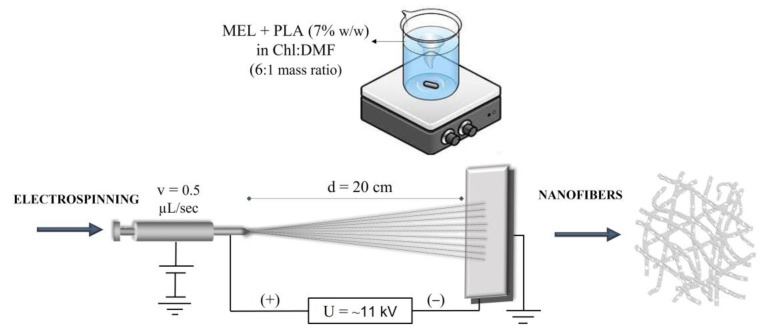
Schematic illustration of the production of melatonin-loaded PLA nanofibers.

The mixtures were stirred at room temperature until a clear viscous solution was obtained. The appropriate flow rate for electrospinning the drug blended PLA polymer solutions was set at 0.5 µL/s and the voltage was adjusted ~11 kV. Figure 1 schematically illustrates the fabrication of MEL-loaded PLA nanofibers.

### 2.3. Preparation and Electrospinning of PVA Nanofibers

A PVA polymer concentration of 12% (*w*/*w*) was used to obtain empty-PVA nanofibers. To improve the solubility in distilled water and to disrupt the strong intra- and interchain bonding of PVA polymer, the PVA solutions were stirred on a magnetic plate and heated to about 80 °C. When a clear viscous solution was obtained the polymeric solution was electrospun at a flow rate 0.1 µL/sec and applied voltage was 15–20 kV. The nanofibers were obtained using the eSpin Cube machine (SpinSplit Ltd., Budapest, Hungary), and were collected in aluminum foil placed at a fixed distance between the needle and the collector of 12.5 cm. The fabrication of MEL-loaded PVA nanofibers was conducted as described below. A water/ethanol solution (1:1, *v/v*) was used to promote MEL solubilization. MEL-loaded PVA nanofibers were obtained by adding MEL solution at different concentration (0.1, 0.3 and 0.5% *w*/*w*) into the PVA polymer solution with 12% *w*/*w* PVA concentration, in presence or absence of permeation enhancer Tween^®^ 80 (0.5% *w*/*w*). The composition and quantity of the components applied for the preparation of the PVA-based nanofiber samples are listed in Table 2.

**Figure 2:**
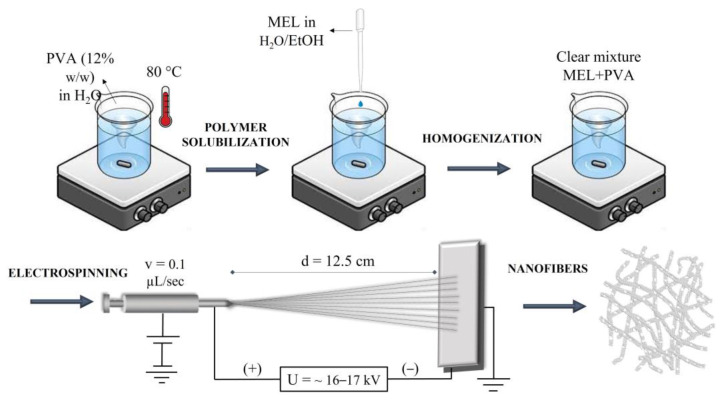
Schematic illustration of the production of melatonin-loaded PVA nanofibers.

The mixture was stirred at room temperature until a homogeneous, clear viscous solution was obtained. The applied voltage for electrospun MEL blended PVA polymer solution was ~16–17 kV. The schematic illustration of the electrospinning process was shown in Figure 2.

### 2.4. Scanning Electron Microscopy

The electrospun samples prepared on aluminum foil were fixed by a conductive double-sided carbon adhesive tape and after that were coated with a gold layer (JEOL JFC-1200 Fine Coater, JEOL Ltd., Tokyo, Japan). SEM images were taken with JEOL JSM-6380LA scanning electron microscope (JEOL Ltd., Tokyo, Japan). The measurements were performed at an acceleration voltage of 15 kV and a working distance of 10 mm. Diameters of 100 individual fibers were measured with ImageJ software (US National Institutes of Health). From the data collected, the average diameter, the standard deviation (SD) and the nanofibers diameter distribution were calculated.

### 2.5. Thermal Characterization of Nanofibers

The thermal properties of the empty and MEL-loaded nanofibers with and without Tween^®^ 80 and of the raw materials were evaluated by differential scanning calorimetry using a thermal analysis instrument model DSC1 Star System (Mettler Toledo, Schwerzenbach, Switzerland) provided of a Poly-Science thermoregulator (PolyScience, Niles, IL, USA). The detection system consisted of high-sensitivity HSS8 and FRS56 ceramic detectors with 120 and 56 thermocouples respectively. For the DSC analysis, 5 mg samples were weighed and sealed with a sealing machine in 40 μL aluminum crucible. The samples were heated in a temperature range of 20–250 °C with a scanning speed of 5 °C/min. Mettler STARe Evaluation system software installed on DELL Optiplex3020 was used for data collection.

### 2.6. FT-IR Spectroscopy Measurements

Pure MEL, pure polymers, Tween^®^ 80, physical mixture (PhM) between MEL and pure polymer (MEL + PVA and MEL + PLA) and all nanofiber samples were analyzed using a Jasco FT/IR-4200 spectrophotometer. The instrument was equipped with single reflection accessory (Jasco ATR PRO470-H) detector. The measurements were detected in absorbance mode and background was acquired before of each measure. For each spectrum, 100 scans were collected over the range of 4000 and 500 cm^−1^, at a resolution of 4 cm^−1^ at room temperature. Spectra Manager-II software (Jasco, Easton, MD, USA) was used for data acquisition.

### 2.7. In Vitro Drug Release Study

The dissolution test was performed by modifying a previously described technique based on the basket method reported by Pharmacopoeia Hungarica (Ph.Hg. VIII) [25]. The in vitro release behavior of MEL-loaded nanofibers with and without Tween^®^ 80 was evaluated in PBS at pH 7.4. Nanofibers samples removed from the aluminum foil were coiled on a magnetic stirrer and placed in a 4 mm-diameter steel coil to keep them at the bottom of the beaker (inner diameter 23 mm). To detect a concentration of 3 µg/mL, 30, 10 and 6 mg were weighed from the samples containing 0.1, 0.3 and 0.5% (*w*/*w*) of MEL, respectively. The temperature of the medium was adjusted at 37 ± 0.5 °C, and 10 mL of medium was added to the sample which was stirred at 100 rpm. An in-line probe was immersed in the beaker and MEL release profile was monitored at a λ_max_ of 278 nm through spectrophotometry (Jasco-V-750 UV-VIS spectrophotometer) until complete dissolution of the nanofibers. The amount of MEL dissolved was determined by a calibration curve according to a partially validated spectrophotometric method. Release study was performed in triplicate for each formulation. The release curve was obtained from the mean and SD of 3 different batches.

To explore the release kinetics of MEL, the Weibull model was fitted to the dissolution curves according to the following equation:

(1) $$M_{t}=M_{\infty }\left(1-e^{-\frac{({t-t_{0})}^{\beta }}{\tau_{d}}}\right)$$

where M_t_ and M_∞_ indicate the amount of drug released at time t and the maximum amount of drug released respectively. The parameters t_0_ and τ_d_ indicate the lag time and average dissolution time. The shape parameter, denoted as β, characterizes the type of release curve. In detail β = 1 outlines first-order kinetics with an exponential curve, β > 1 shows a sigmoidal curvature, and β < 1 denotes a parabolic curve [26].

### 2.8. Swelling Properties

The swelling behavior of nanofibers was performed using a gravimetric method at room temperature. 10 mg of sample was placed in an aqueous medium (PBS pH 5.5 and 7.4). After 24 h, nanofibrous inserts were removed from the medium, rinsed with distilled water and the excess water was wiped off with paper towels before weighing the samples. The swelling degree (%SD) was calculated according to following equation:

(2) $$\%SD=\frac{W_{s}-W_{d}}{W_{s}}\times 100$$

where W_s_ is the weight of the sample after swelling and W_d_ represents the initial dry weight of the nanofibers. Each experiment was performed in triplicate and the results represent the mean ± SD.

## 3. Results and Discussion

PLA and PVA nanofibers with different concentrations of MEL and with or without the addition of Tween^®^ 80 were prepared by electrospinning to obtain the above samples (Table 1 and Table 2).

PVA is a hydrophilic semi-crystalline polymer that possesses properties of high mechanical strength and excellent electroconductivity. The hydroxyl groups on the side chains of PVA allow the polymer to self-crosslink in aqueous solutions to form soft, flexible but at the same time resistant hydrogels. Due to these unique properties and the recognized biodegradability and biocompatibility, PVA hydrogels have been widely used to produce nanofibers by electrospinning [27]. PVA polymer is available in a variety of molecular weights (MW) and degrees of hydrolysis (DH) that influence its chemical identity. It was reported that the average diameter of nanofibers obtained from PVA increased with increasing DH. PVA with DH equal to 88% ensured the production of fibers with smaller average diameters (∼190 nm) than fibers produced from completely hydrolyzed PVA whose diameters measured 470 nm [28]. With regard to MW, PVA with low (67 kDa), medium (130 kDa), and high (146–186 kDa) MWare available. The viscosity of PVA solutions increased proportionally with increasing MW and the concentration of the polymer solution. Thus, the intrinsic polymeric solution properties determine the success or failure of the electrospinning process. Polymeric solutions at too low or high concentrations and MW resulted in unstable jets, while PVA at medium MW and intermediate concentrations (12% *w*/*w*) improved the stability of the Taylor cone and produced smooth, uniform and bead-free fibres [29].

PLA polymer is synthesized from renewable sources and possesses attractive manufacturing properties [30]. Its high mechanical strength, low cost and electrospinning ability make it an excellent polymer to produce nanofibers [31]. The hydrophobic nature of PLA results in slow erosion in a physiological environment, and this feature has been exploited to produce nanocomposite fibers with prolonged and sustained drug release profiles [32]. In addition to concentration, the choice of solvent or solvent mixture system in which to dissolve the polymer plays a critical role in affecting the electrospinning ability of PLA solutions, as parameters such as surface tension, dielectric constant and boiling point of the solvents have a crucial impact on the process [33]. It has been previously reported that concentrations of no lower than 7% (*w*/*w*) and the use of a solvent mixture system such as Chl:DMF (in a 6:1 mass ratio) could result in continuous and bead-free nanofibrous structures [34]. To further improve the physical properties of ocular implants the addition of Tween^®^ 80 was investigated.

### 3.1. Morphological Characterization

**Figure 3:**
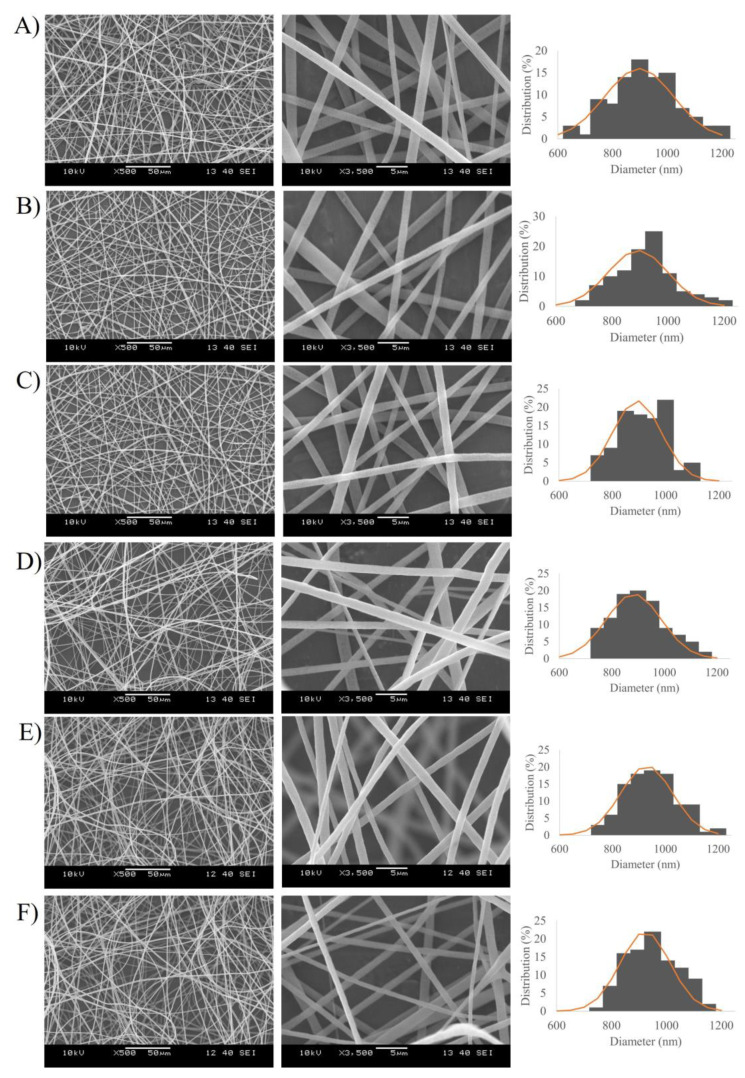
SEM images (magnification: 500× and 3500×) and diameter distribution of Melatonin-loaded PLA nanofibers with different concentration of drug 0.1% *w*/*w* (**A**), 0.3% *w*/*w* (**B**), 0.5% *w*/*w* (**C**), and Melatonin-loaded PLA nanofibers with Tween^®^ 80 and different concentrations of drug 0.1% *w*/*w* (**D**), 0.3% *w*/*w* (**E**), 0.5% *w*/*w* (**F**).

**Figure 4:**
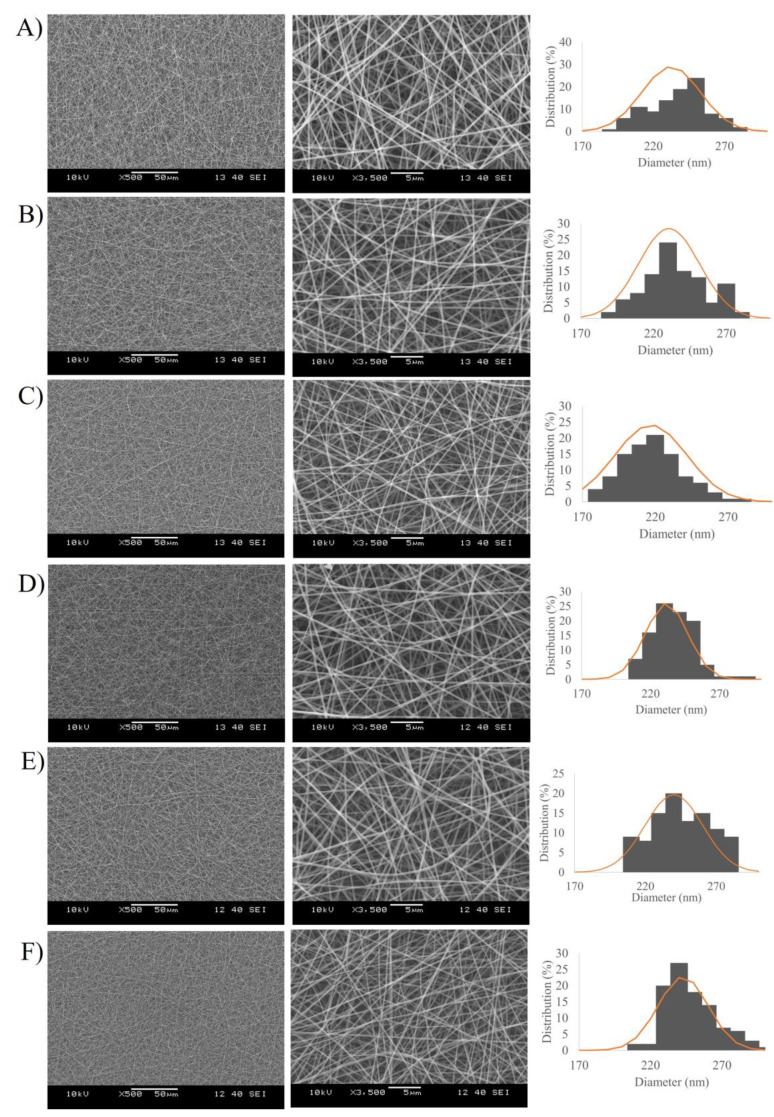
SEM images (magnification: 500× and 3500×) and diameter distribution of Melatonin-loaded PVA nanofibers with different concentration of drug 0.1% *w/w* (**A**), 0.3% *w/w* (**B**), 0.5% *w/w* (**C**), and Melatonin-loaded PVA nanofibers with Tween^®^ 80 and different concentrations of drug 0.1% *w/w* (**D**), 0.3% *w/w* (**E**), 0.5% *w/w* (**F**).

The morphological properties of the prepared nanofibers were observed by SEM analysis. The SEM images and fiber diameter distribution graph of the electrospun PLA and PVA nanofibers are shown in Figure 3 and Figure 4, respectively. It was clearly demonstrated that the nanofibers were successfully obtained via electrospinning of the polymer solutions. PLA-based samples (Figure 3) showed randomly oriented fibrous structures with rough surfaces, no beads, and submicron fiber diameters ranging from 883 ± 106.90 to 923.08 ± 90.71 nm. From Figure 4, the PVA nanofibers had a smooth and uniform morphology without bead formation, with average diameters between 217.03 ± 24.77 and 242.83 ± 17.50 nm. No noticeable variations in nanofiber diameter were found to be dependent on the loading of different concentrations of MEL or the presence of Tween^®^ 80.

### 3.2. Differential Scanning Calorimetry Measurements

**Figure 5:**
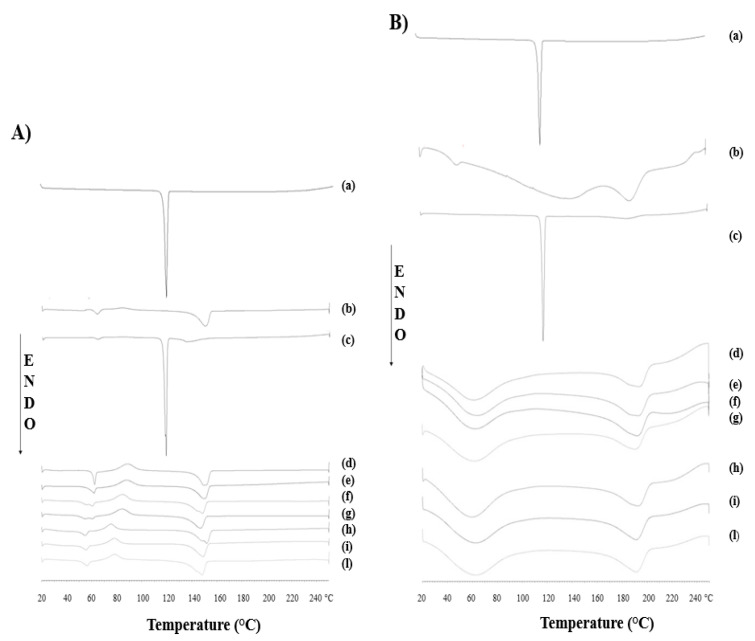
DSC curves of (**A**) Melatonin (a), PLA polymer (b), physical mixture MEL + PLA polymer (c), empty-PLA nanofibers (d), Melatonin-loaded PLA nanofibers with different concentration of drug 0.1% *w/w* (e), 0.3% *w/w* (f), 0.5% *w/w* (g), and Melatonin-loaded PLA nanofibers with Tween^®^ 80 and different concentration of drug 0.1% *w/w* (h), 0.3% *w/w* (i), 0.5% *w/w* (l); and (**B**) Melatonin (a), PVA polymer (b), physical mixture MEL+PVA polymer (c), empty-PVA nanofibers (d), Melatonin-loaded PVA nanofibers with different concentration of drug 0.1% *w/w* (e), 0.3% *w/w* (f), 0.5% *w/w* (g), and Melatonin-loaded PVA nanofibers with Tween^®^ 80 and different concentration of drug 0.1% *w/w* (h), 0.3% *w/w* (i), 0.5% *w/w* (l).

To investigate the thermal behavior of the nanofibers, DSC measurements were carried out. The heating curves of the electrospun samples and starting materials are shown in Figure 5, in detail the thermograms of the PLA-based nanofibers are collected in graph (A), those of the PVA-based nanofibers in graph (B). A distinct melting endotherm was evident in the curves (a) of both graphs at 118 °C, which corresponded to the characteristic melting peak of MEL. Concerning Figure 5A, the PLA polymer thermogram (curve (b)) showed the typical characteristics of semi-crystalline thermoplastics material with three distinct events: the first endothermic-type event at 64 °C indicated the glass transition temperature (T_g_), the second exothermic-type event at 85 °C consisted of the crystallization phase, and finally the melting peak (T_m_) at 150 °C [35]. In the PhM between MEL and neat PVA (curve c), the thermal behavior of the individual components was observed; the drug peak remained unchanged, while the polymer thermal events showed negligible shifts, where T_g_ of the polymer shifted to slightly higher values (69 °C), and the melting peak decreased about 15 °C. Regarding the thermograms of all PLA-based nanofibrous scaffolds, no characteristic MEL peak was observed, suggesting that the drug was in amorphous phase within the electrospun nanofibers. The reason could be attributed to rapid solvent evaporation during the electrospinning process, which could prevent the drug molecules to form crystalline lattices [36].

Furthermore, it was observed that the incorporation of the surfactant Tween 80^®^ into the structure modified the crystallinity conditions of the nanofibers. The scaffolds producing with surfactant (curves h, i, and l) showed a 10 °C reduction in both the T_g_ (from 85 to 75 °C) and the crystallization phase of the polymer (from 64 to 54 °C). This reduction could be attributed to the plasticizing effect of the permeation enhancer, which would increase the free volume within the polymer matrix, improve the mobility of the polymer chains that could lead to higher scaffold elasticity [37,38].

Regarding the graph in Figure 5B, two characteristic endothermic events of PVA were observed in the thermogram of the polymer at 45 and 188 °C, indicating T_g_ and T_m_, respectively. In the PhM between PVA and MEL (curve b), the peaks of the individual constituents were present. All the curves of the PVA-based nanofibers, empty and loaded with MEL, with and without the surfactant, showed a similar trend. Again, the MEL peak was not observed in any of the thermograms, confirming the presence of the drug in the amorphous state, while the polymer peaks were observed at higher temperatures, specifically the T_g_ was shifted from 45 to 65 °C and the T_m_ from 188 to 193 °C. The higher heat demand required to melt the nanofibers could suggest that the electrospinning process promoted a more ordered and confined orientation of the polymer chains.

### 3.3. FT-IR Analysis

**Figure 6:**
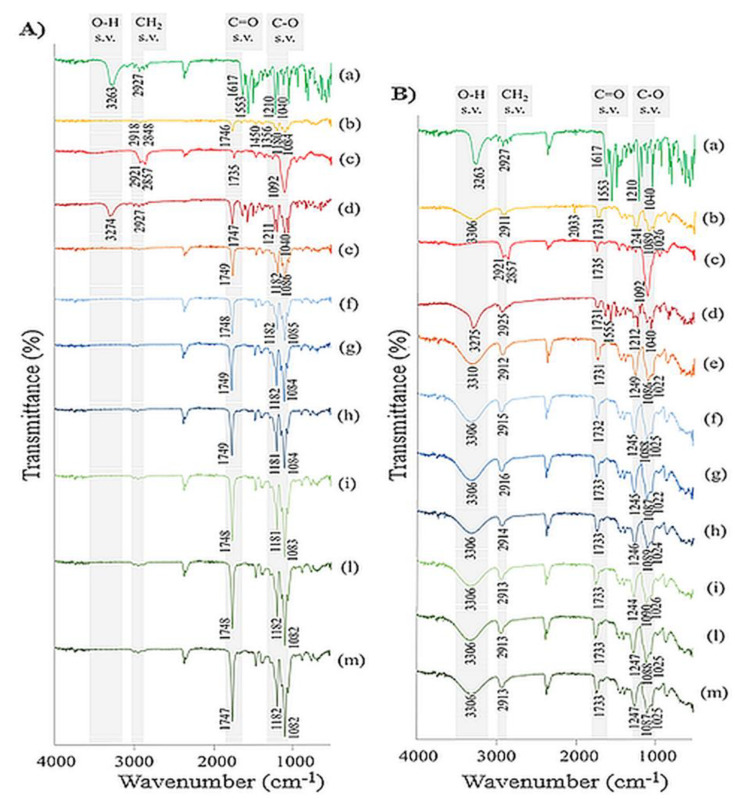
FT-IR curves of (**A**) Melatonin (a), PLA polymer (b), Tween^®^ 80 (c), physical mixture MEL+PLA polymer (d), empty-PLA nanofibers (e), Melatonin-loaded PLA nanofibers with different concentration of drug 0.1% *w/w* (f), 0.3% *w/w* (g), 0.5% *w/w* (h), and Melatonin-loaded PLA nanofibers with Tween^®^ 80 and different concentration of drug 0.1% *w/w* (i), 0.3% *w/w* (l), 0.5% *w/w* (m); and (**B**) Melatonin (a), PVA polymer (b), Tween^®^ 80 (c), physical mixture MEL+PVA polymer (d), empty-PVA nanofibers (e), Melatonin-loaded PVA nanofibers with different concentration of drug 0.1% *w/w* (f), 0.3% *w/w* (g), 0.5% *w/w* (h), and Melatonin-loaded PVA nanofibers with Tween^®^ 80 and different concentration of drug 0.1% *w/w* (i), 0.3% *w/w* (l), 0.5% *w/w* (m).

Pure components, physical mixtures and electrospun nanofibers were investigated by FT-IR spectroscopy to assess potential molecular interactions and to confirm the chemical composition of the composite nanofibers (Figure 6). MEL showed the N-H stretching and bending at 3263 and 1553 cm^−1^, respectively, the vibration peak for C=O amidic carbonyl group was identified at 1617 cm^−1^ and the characteristic stretching signals for C–N group were detected at 1210 and 1040 cm^−1^ [39]. PVA polymer spectra showed absorption band related to O-H stretching vibration between 3500–3200 cm^−1^, the peaks in the 3000–2800 cm^−1^ region were attributed to the CH_2_ alkyl groups, the stretching peak assigned for C=O was identified at 1731 cm^−1^, the peaks at 1026 and 1089 cm^−1^ represented the C–O stretching vibration and the C–H bending and deformations were identified in the finger print region (900–500 cm^−1^). Similar results were reported by other authors [40,41]. The FT-IR spectrum of pure PLA showed the characteristic absorption bands for C–H alkane group vibration at 2918 and 2848 cm^−1^, the peak at 1746 cm^−1^ was assigned to C=O stretching, the C–H alkane groups bending vibrations were observed at 1450 and 1356 cm^−1^ and the bands identified between 1210 and 1040 cm^−1^ were assigned to C–O stretching vibration [42]. For Tween^®^ 80 the broad band at 3500 cm^−1^ was attributed to the O–H stretching vibration, the absorption band between 2921 and 2857 cm^−1^ reflected the C–H stretching of the methylene groups, the stretching band for C=O was observed at 1735 cm^−1^ and the narrow peak at 1092 cm^−1^ was related to C–O–C stretching. Concerning the spectrum of PhM between MEL and PVA, the high-frequency regions were similar to the MEL scan, with a narrow peak at 3275 cm^−1^ for N–H stretching and the region between 3000–2800 cm^−1^ identified for CH_2_ alkyl groups. The low-frequency region was similar to pure PVA polymer, with bands attributable to C=O stretching (1731 cm^−1^) and C–O stretching (1000–1200 cm^−1^). Similar results were observed for PhM containing the PLA polymer, in which the low-frequency region showed an absorption peak at 1747 cm^−1^ for carbonyl C=O and C–O stretching vibrations between 1210 and 1040 cm^−1^. No difference was observed for all PVA nanofibers scans (empty, loaded with different concentrations of MEL and with or without Tween^®^ 80). In detail, the spectra showed no peak attributable to MEL, but rather similarities with the spectrum of neat polymer. The absorption band between 3500–3200 cm^−1^ appeared broader compared to PVA alone, the C=O carbonyl group stretching, and the C–O stretching bands were detected at a higher intensity of both polymer and PhM. Also, for the PLA nanofibers, no differences were found between the spectra, all of which showed an overlapping trend with the absence of the typical absorption peaks of MEL. Similarly, for the bands attributable to the C=O and C–O groups observed in all samples and representing the characteristic peaks of the PLA polymer, more intense absorption peaks were identified compared to the PhM and the raw polymer. The higher intensity that was observed for C=O and C–O stretches in both types of nanofibers could suggest the formation of hydrogen bonds that probably occurred during the polymer dissolution phase. It has been demonstrated that polymer solubility in polar solvents is dominated by interactions such as hydrogen bonds. The results of FT-IR analysis proved that PVA and PLA nanofibers were successfully fabricated.

### 3.4. In Vitro Drug Release from the Nanofibers

The two nanofibers showed different release profiles according to their nature.

**Figure 7:**
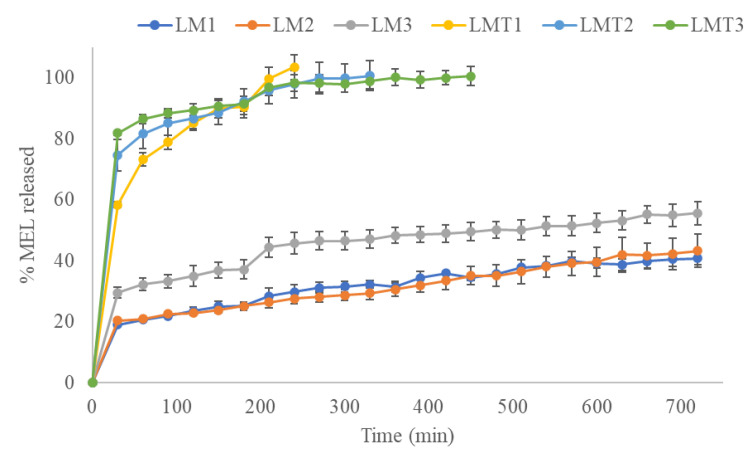
In vitro release profiles of MEL-loaded PLA nanofibers Melatonin-loaded PLA nanofibers with different concentration of drug 0.1% *w/w* (LM1), 0.3% *w/w* (LM2), 0.5% *w/w* (LM3), and Melatonin-loaded PLA nanofibers with Tween^®^ 80 and different concentration of drug 0.1% *w/w* (LMT1), 0.3% *w/w* (LMT2), 0.5% *w/w* (LMT3) in phosphate buffered solution (pH 7.4) at 37 °C. Each point represents the mean value of three different experiments ±S.D.

The PLA-based nanofibers exhibited slow drug release, and the saturation curves of scaffolds prepared with and without Tween^®^ 80 showed substantial differences (Figure 7). The nanofibers containing Tween^®^ 80 released the full amount of encapsulated melatonin within 4, 5, and 6 h for drug concentrations of 0.1, 0.3, and 0.5% (*w/w*), respectively. In contrast, nanofibers produced in the absence of Tween^®^ 80 showed sustained release; after 12 h percentages of melatonin between 40 and 55% were released from the scaffolds.

**Figure 8:**
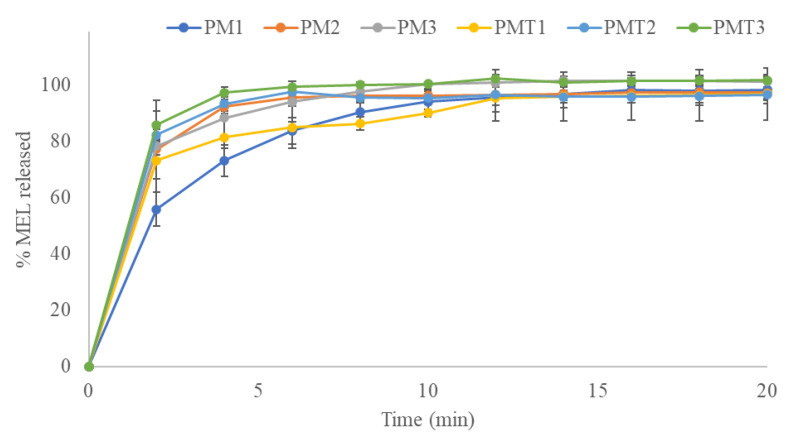
In vitro release profiles of MEL-loaded PVA nanofibers Melatonin-loaded PVA nanofibers with different concentration of drug 0.1% *w/w* (PM1), 0.3% *w/w* (PM2), 0.5% *w/w* (PM3), and Melatonin-loaded PVA nanofibers with Tween^®^ 80 and different concentration of drug 0.1% *w/w* (PMT1), 0.3% *w/w* (PMT2), 0.5% *w/w* (PMT3) in phosphate buffered solution (pH 7.4) at 37 °C. Each point represents the mean value of three different experiments ± S.D.

In contrast, nanofibers composed of PVA released the entire MEL load within 20 min (Figure 8); the release profiles of the fibers produced with or without Tween^®^ 80 showed overlapping curves.

The faster release from PVA nanofibers could be explained by the interaction of the hydrophilic polymer with the water molecules in the release medium [28]. The interaction of the hydroxyl groups of PVA in contact with the water molecules results in the dissolution of the fibers and therefore a fast amount of drug could be released. Conversely, hydrophobic PLA has a weak interaction with water molecules, resulting in a slower release profile [43].

The remarkable difference in the release profiles of PLA fibers with or without Tween^®^ 80 could presumably be attributed to the porosity of the nanofibers prepared with the addition of permeation enhancer. In fact, Tween^®^ 80 could result in the formation of fibers with a more open structure that speeds up melatonin release compared with fibers obtained without the surfactant.

The Weibull model was employed to characterize the release kinetics of MEL from nanofibrous samples. The release kinetic parameters of the examined scaffolds are listed in Table 3. The shape parameter of curve (β) was scored for all samples >1 indicating a sigmoidal trend. The MEL release rate was characterized by an initial almost exponential increase, followed by an asymptotic slowdown in which no more growth in drug release was observed, so that the curve assumed a linear trend [44]. This sigmoidal curve could be attributed to the penetration of water between the nanofibers and the subsequent MEL dissolution in the aqueous medium; therefore, diffusion could be the governing factor which generates this release rate [45].

All correlation coefficients of the linear regression analysis showed values >0.9, suggesting a satisfactory fit of the collected data to the Weibull model.

### 3.5. Swelling Properties in Aqueous Media

**Figure 9:**
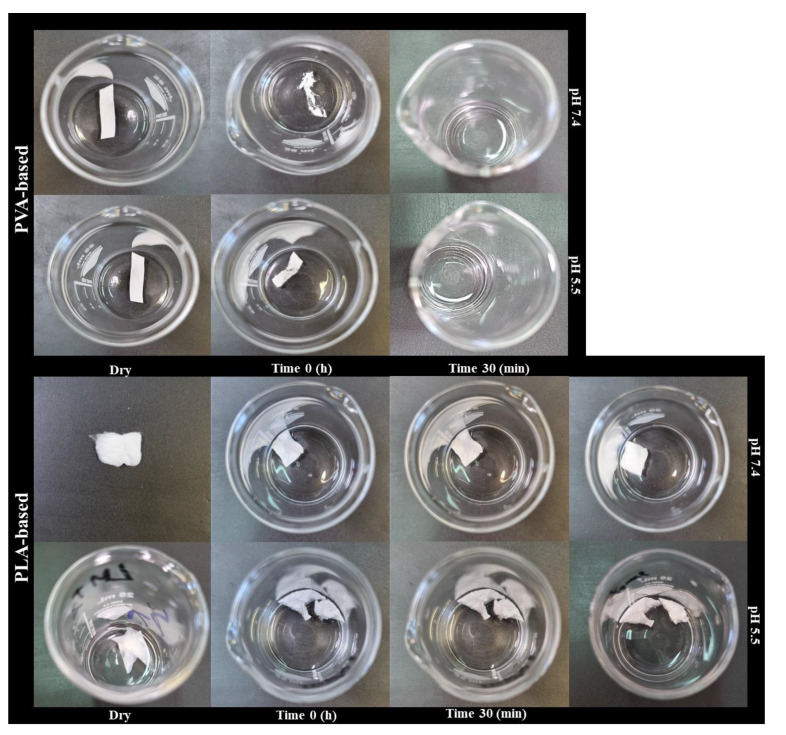
Images of the PVA (top) and PLA-based nanofibres (bottom) in the dry state, after addition of the medium (time 0), after 30 min and at the end of the experiment (24 h) at the two pH conditions tested (5.5 and 7.4).

The swelling behavior of nanofiber was determined at pH 5.5 and 7.4 within 24 h. Pictures of the samples in the dry state, after medium addition (time 0), after 30 min and at the end of the experiment (24 h) were collected and shown in Figure 9.

**Figure 10:**
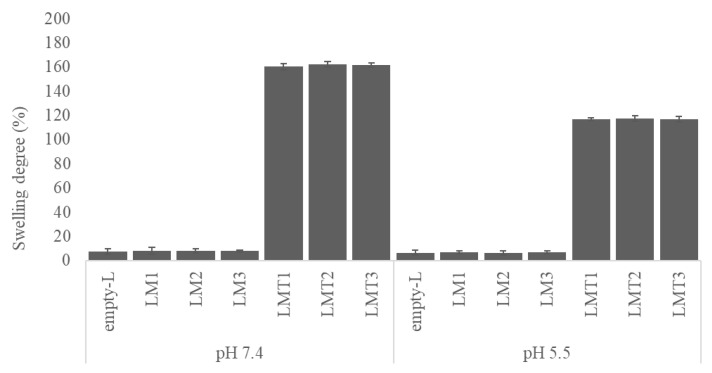
Swelling behavior of electrospun PLA-based nanofibers at pH 7.4 and 5.5.

According to the results of the in vitro release test, the hydrophilic PVA-based nanofibers showed complete dissolution in the aqueous medium within 30 min. For these samples, the experiment was stopped at this time and %SD data, although experimentally observed were not reported. Figure 10 shows that the swelling rate of nanofibers is much higher in the scaffold prepared with the addition of hydrophilic permeation enhancer. After 24 h, a swelling degree of 8% and 6% was measured for all purely PLA-based nanofibers at pH 7.4 and 5.5, respectively. The addition of the permeation enhancer showed an increase in swelling around 20-fold, reaching values of 160% and 117% at pH 7.4 and 5.5. These results confirm what was found in MEL release profiles, i.e., the addition of the hydrophilic component in the hydrophobic polymer matrix promotes greater water uptake, swelling and thus faster drug diffusion [46].

Concerning the different aqueous media %SD was higher in pH 7.4 than in pH 5.5 of all the studied samples. Swelling of samples decreased from 160% to 117% and from 8% to 6% in pH 7.4 and 5.5, respectively. The degradation of PLA is strongly influenced by pH and acidic conditions compared to physiological levels resulted in a slower degradation rate [47,48].

## 4. Conclusions

In this study, the electrospinning technique was successfully used to produce MEL-loaded nanofibers. SEM studies confirmed the nanofibrous structure, while thermal and spectroscopic characterization of the samples confirmed that the neat crystalline MEL was in the amorphous form in the scaffolds. According to the Weibull model, the release kinetics followed a sigmoidal pattern, suggesting diffusive processes for MEL. Although, drug release profiles from nanofibers indicated different dissolution rates according to the nature of the polymer. Hydrophilic PVA nanofibers showed an immediate within 20 min and complete MEL releases in contrast to the hydrophobic PLA ones. An interesting finding was that Tween^®^ 80 addition in PLA-based nanofibers provided a faster dissolution, a complete release profile and an approximately 20-fold increase in swelling properties, suggesting a probable structural improvement of nanofibrous inserts. Based on the obtained results, the formulated MEL-loaded nanofibers could represent a promising vehicle with improved biopharmaceutical characteristics for the ocular delivery of MEL. Further in vivo studies are required to approve the biological activity of the absorbed melatonin.

## Figures and Tables

**Table 1:** Composition of PLA-based nanofiber samples.

Sample	Melatonin (% *w*/*w*)	Tween^®^ 80 (% *w*/*w*)
Empty-L	0	0
LM1	0.1	0
LM2	0.3	0
LM3	0.5	0
LMT1	0.1	0.5
LMT2	0.3	0.5
LMT3	0.5	0.5

**Table 2:** Composition of PVA-based nanofiber samples.

Sample	Melatonin (% *w*/*w*)	Tween^®^ 80 (% *w*/*w*)
Empty-P	0	0
PM1	0.1	0
PM2	0.3	0
PM3	0.5	0
PMT1	0.1	0.5
PMT2	0.3	0.5
PMT3	0.5	0.5

**Table 3:** Dissolution kinetic parameters of Melatonin-loaded PLA and PVA nanofibers.

Sample	β Parameter	τ_d_	Correlation Coefficient
LM1	5.0607	35.22	0.9612
LM2	4.8539	34.81	0.9483
LM3	6.1983	49.53	0.9367
LMT1	5.8622	91.42	0.9792
LMT2	11.4432	95.11	0.9737
LMT3	17.1458	97.40	0.9279
PM1	5.2927	93.32	0.9609
PM2	8.0208	93.10	0.9157
PM3	10.3280	95.83	0.9717
PMT1	11.2443	91.67	0.9631
PMT2	10.7446	94.78	0.9677
PMT3	12.7788	99.11	0.9286

## Data Availability

The data presented in this study are available on request from the corresponding author.
